# Biofabrication of functional protein nanoparticles through simple His-tag engineering

**DOI:** 10.1021/acssuschemeng.1c04256

**Published:** 2021-08-30

**Authors:** Hèctor López-Laguna, Julieta M. Sánchez, José Vicente Carratalá, Mauricio Rojas-Peña, Laura Sánchez-García, Eloi Parladé, Alejandro Sánchez-Chardi, Eric Voltà-Durán, Naroa Serna, Olivia Cano-Garrido, Sandra Flores, Neus Ferrer-Miralles, Verónica Nolan, Ario de Marco, Nerea Roher, Ugutz Unzueta, Esther Vazquez, Antonio Villaverde

**Affiliations:** † Institut de Biotecnologia i de Biomedicina, Universitat Autònoma de Barcelona, Bellaterra, Barcelona 08193, Spain; ‡ Departament de Genètica i de Microbiologia, Universitat Autònoma de Barcelona, Bellaterra, Barcelona 08193, Spain; § CIBER de Bioingeniería, Biomateriales y Nanomedicina (CIBER-BBN), C/Monforte de Lemos 3-5, Madrid 28029, Spain; ∥ Universidad Nacional de Córdoba, Facultad de Ciencias Exactas, Físicas y Naturales, ICTA and Departamento de Química, Cátedra de Química Biológica, Av. Vélez Sársfield 1611, Córdoba 5016, Argentina; ⊥ CONICET-Universidad Nacional de Córdoba, Instituto de Investigaciones Biológicas y Tecnológicas (IIByT), Av. Velez Sarsfield 1611, Córdoba, 5016, Argentina; # Servei de Microscòpia, Universitat Autònoma de Barcelona, Bellaterra, Barcelona 08193, Spain; ∇ Departament de Biologia Evolutiva, Ecologia i Ciències Ambientals, Facultat de Biologia, Universitat de Barcelona, Av. Diagonal 643, Barcelona 08028, Spain; ○ Laboratory for Environmental and Life Sciences, University of Nova Gorica, Nova Gorica 5000, Slovenia; ● Biomedical Research Institute Sant Pau (IIB Sant Pau), Sant Antoni Ma Claret 167, Barcelona 08025, Spain; ▲ Departament de Biologia Cel·lular, Fisiologia Animal i Immunologia, Universitat Autònoma de Barcelona, Barcelona 08193, Spain

**Keywords:** Nanoparticles, Protein engineering, Divalent cations, Protein materials, Biomaterials design

## Abstract

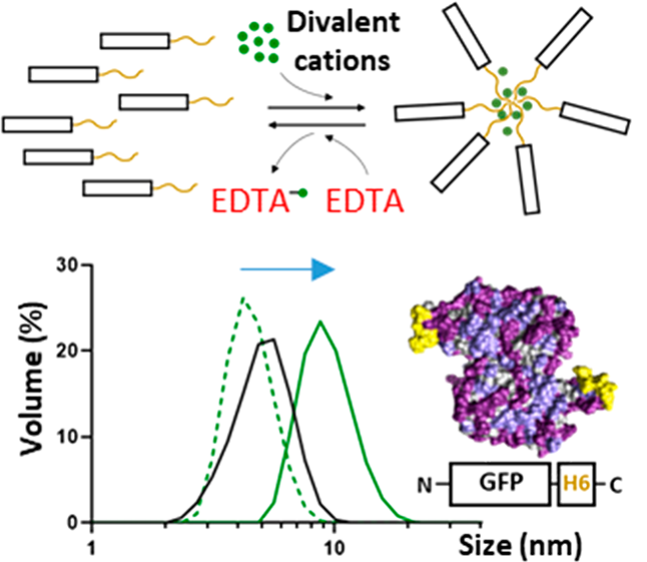

We have developed a simple, robust, and fully transversal approach for the *a-la-carte* fabrication of functional multimeric nanoparticles with potential biomedical applications, validated here by a set of diverse and unrelated polypeptides. The proposed concept is based on the controlled coordination between Zn^2+^ ions and His residues in His-tagged proteins. This approach results in a spontaneous and reproducible protein assembly as nanoscale oligomers that keep the original functionalities of the protein building blocks. The assembly of these materials is not linked to particular polypeptide features, and it is based on an environmentally friendly and sustainable approach. The resulting nanoparticles, with dimensions ranging between 10 and 15 nm, are regular in size, are architecturally stable, are fully functional, and serve as intermediates in a more complex assembly process, resulting in the formation of microscale protein materials. Since most of the recombinant proteins produced by biochemical and biotechnological industries and intended for biomedical research are His-tagged, the green biofabrication procedure proposed here can be straightforwardly applied to a huge spectrum of protein species for their conversion into their respective nanostructured formats.

## Abstract 2

A simple, cost-effective, and green method is proposed to generate functional, protein-only nanoparticles with clinical applicability out of any desired polypeptide.

## Introduction

Protein materials result from the controlled self-assembly of individual polypeptides under defined architectural patterns.^1,2^ In contrast to other chemical categories of building blocks, proteins generate supramolecular materials that benefit from the structural and functional capabilities and the particular versatility shown by these biomolecules. Especially, their intrinsic biocompatibility and biodegradability offer a wide applicability in clinics^3−5^ over alternative, potentially toxic materials such as metals, polymers, ceramics, and lipids. The interdependence between structure and function makes protein-based materials particularly appealing, since these properties can be tuned by rational genetic engineering. The formation of supramolecular protein materials out of self-assembled building blocks is common in nature. For instance, recombinant silk proteins,^6,7^ viral protomers,^8^ or amyloid peptides^9−11^ self-organize into complex oligomeric structures because of the natural tendency of the building blocks to specifically cross-interact into defined geometries, within nano- or microscales. More complicated is conferring, on purpose, self-assembling abilities to clinically relevant polypeptides, which being intrinsically monomeric might be appealing as functional, more complex multimeric materials. In particular, innovative medicines demand biologically safe nanoscale materials that, being biologically active, might assist in a diversity of clinical fields. Cross-molecular interactions can be conferred by self-assembling protein domains recruited from nature; when they are fused to a protein of interest, they drive the organization of the whole fusion into regular oligomers.^12,13^ Of course, diverse categories of chemical cross-linkers have also been explored for the construction of protein materials.^14−16^ However, the design of materials usable in biological interfaces should be ideally achieved through self-assembly, with a minimal extent of protein engineering and without the addition of chemical couplers that are potentially toxic. This would minimize perturbations of the original protein conformation, preserve the full functionality, and avoid the use of hazardous and recalcitrant chemicals or the application of poorly green processes.

**Figure 1:**
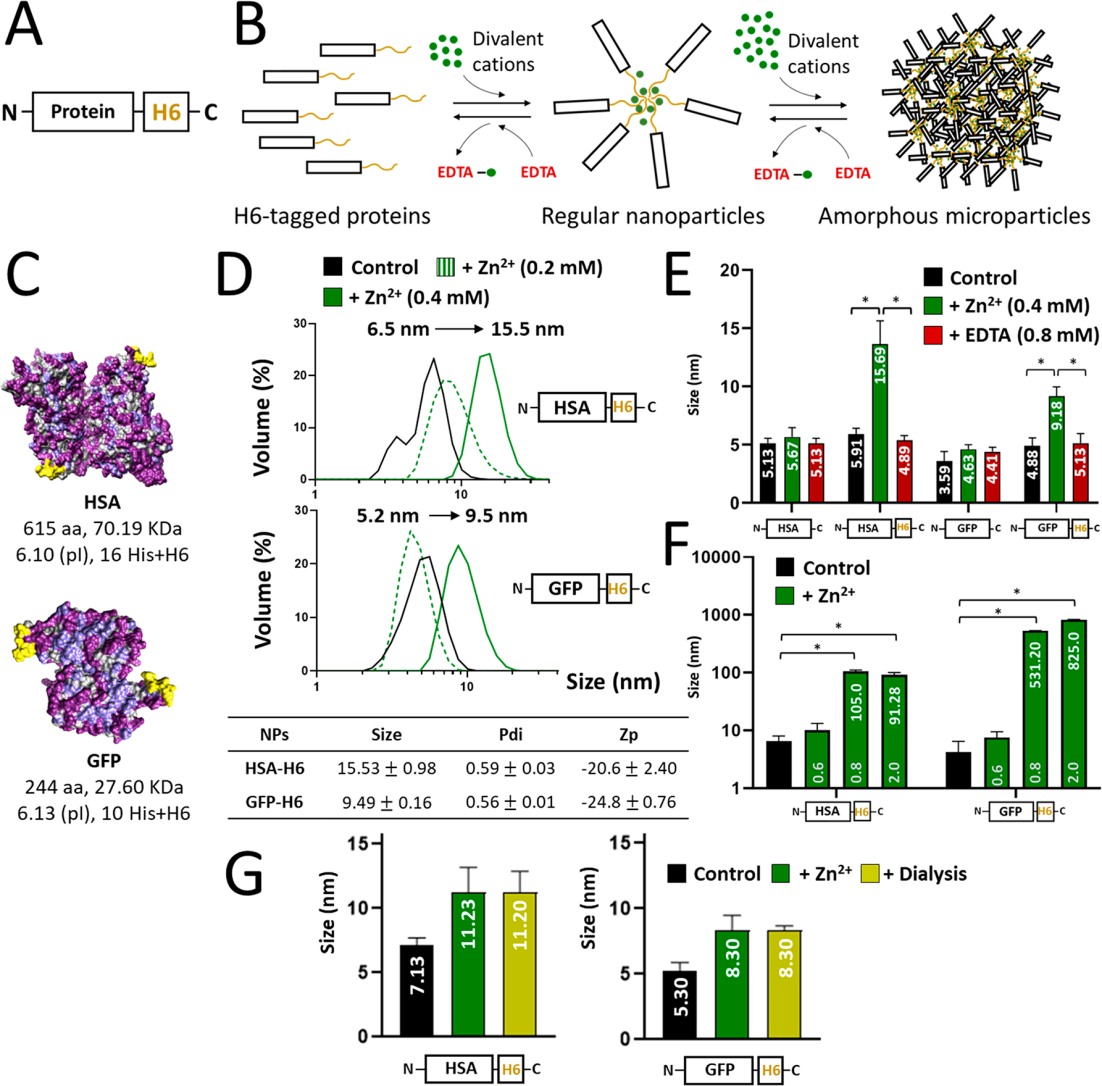
Proposed concept of progressive protein oligomerization supported by divalent cations and self-assembly potential of H6-tagged HSA and GFP. (A) Schematic representation of polyhistidine-tagged proteins. A histidine-rich peptide, usually the hexahistidine H6, is fused to the carboxy terminus of any recombinant protein to allow its one-step purification by affinity chromatography. Other humanized His-rich peptides are also available as efficient purification tags.^56^ (B) Progressive assembly of H6-tagged proteins mediated by increasing amounts of divalent cations such as Zn^2+^. The formation of MPs has been experimentally demonstrated,^18,19^ but regular nanoparticles are expected as intermediates in the process. In each step, disassembly can be achieved by using the chemical chelator EDTA. Spontaneous disassembly of MPs has been observed under physiological conditions,^19,82^ supporting the concept that such materials are mimetics of the secretory granules for peptide hormones in the mammalian endocrine system. (C) Dimeric structures (the most plausible building block forms) of HSA and GFP showing the exposed His tags in yellow. Bulk atoms are depicted in gray and cationic and hydrophilic groups in pale and deep purple, respectively. A short summary of the main biochemical properties of each construct is also displayed. (D) Hydrodynamic size (nm) of HSA-H6 and GFP-H6 in the absence and presence of Zn^2+^ at increasing concentrations, determined by DLS. The peak (Size), polydispersion index (Pdi) and Z potential (Zp, expressed in mV), in the presence of 0.4 mM Zn^2+^ are also indicated at the bottom. (E) Role of the H6 tag in protein assembly and EDTA-mediated disassembly. The hydrodynamic sizes in nm are indicated inside the histograms as white numbers. (F) Aggregation of HSA-H6 and GFP-H6 at increasing Zn^2+^ concentrations (0.6, 0.8, and 2 mM). White numbers indicate the hydrodynamic size in nm. Data are expressed as *x̅* ± SEM with at least *n* = 3, and statistical significance is achieved when *p < 0.05. (G) Hydrodynamic size (nm) of Zn^2+^-based HSA-H6 and GFP-H6 NPs extensively dialyzed against a Zn^2+^-free buffer, determined by DLS.

In this context, histidine (His)-rich peptides have been recently revealed as potent and intrinsic protein cross-linkers.^17^ Their coordination with divalent cations such as Zn^2+^ induces protein–protein interactions and clustering as protein-only microparticles.^18,19^ The materials, essentially amorphous and having the size of a few micrometers, mimic the amyloidal architecture of bacterial inclusion bodies^20^ and that of secretory granules from the mammalian endocrine system.^5,21−23^ On this basis, we wondered if the controlled use of divalent cations at doses below those triggering microscale protein aggregation would represent a reliable approach to fabricate regular nanoparticles based on polyhistidine-tagged building block proteins (Figure 1A). We speculated that protein nanoparticles (NPs) might be a category of intermediates in the aggregation process that leads to the formation of amorphous microparticles (MPs, Figure 1B) and that their formation and stability might be controlled by adjusting the concentration of divalent cations in the coordination event. It must be noted that in biotechnology, recombinant proteins are commonly produced as His-tagged fusion constructs, since the short end-terminal polyhistidine stretches (such as the hexahistidine H6) do not abolish protein functionalities but permit their one-step purification by affinity chromatography from cell extracts.^24^ Therefore, divalent cations, used as a molecular glue for His-tagged proteins, would straightforwardly allow the *a-la-carte* fabrication of protein nanoparticles out of most of the recombinant proteins currently available in research and pharma laboratories. This could be done without further engineering and by means of a transversal assembly platform that runs in the absence of xenobiotic cross-linkers.

## Materials and Methods

### Design, Production, and Purification of Protein Constructs

β-Galactosidase (β-Gal), green fluorescent protein (GFP), and human serum albumin (HSA) were provided by Sigma-Aldrich (No, 5635), Roche (No. 11814524001), and Grifols (hemoderivative), respectively. For the other proteins, codon-optimized genes were produced by Geneart (ThermoFisher) and cloned in a pET22b vector (Novagen). Plasmids encoding GFP-H6, STM-H6, H6-GFP-T22, and BAK-GFP-H6 were transformed into *E. coli* Origami B cells (BL21, *ompT*, *lon*, TrxB, Gor^–^; Novagen) and TRX-H6-hLIF into *E. coli* BL21 (DE3; Novagen) cells. A1-, B4-, and F7-GFP-H6 plasmids were transformed into *E. coli* BL21sox cells^2^ by thermal shock at 42 °C for 45 s. Bacterial cells were grown in lysogenic broth (LB) medium, and proteins were produced overnight at 20 °C (GFP-H6, TRX-H6-hLIF, STM-H6, A1-, B4-, and F7-GFP-H6) or 37 °C for 3 h (H6-GFP-T22 and BAK-GFP-H6) upon addition of 0.1 mM isopropyl-β-d-thiogalactopyronaside (IPTG) or 1 mM IPTG (for A1-GFP-H6 production). Cells were subsequently harvested at 5000*g* for 15 min, resuspended in wash buffer (20 mM Tris-HCl, 500 mM NaCl, 10 mM imidazole, pH 8) in the presence of protease inhibitors (cOmplete EDTA-free, Roche Diagnostics), and disrupted by two rounds of 1200 psi in a French Press (Thermo FA-078A). The soluble cell fraction was recovered by centrifugation at 15000*g* for 45 min. On the other hand, a codon-optimized HSA-H6 gene was also synthesized by GeneArt (ThermoFisher Scientific), supplied into a pTriex6 plasmid, and transfected into HEK-293F mammalian cells in the presence of PEI (polyethylenimine, in the ratio 3:1), when the cell concentration reached 10^6^ cells mL^–1^. Cells were then cultured in Freestyle 293 medium and the recombinant protein was produced and excreted to the media due to a secretion peptide, in the presence of valproic acid (4 mM), for 6 days (37 °C, 70% humidity, 8% CO_2_ and 120 rpm). The culture medium containing the secreted soluble protein was finally separated from the cells by centrifugation at 300*g* for 15 min.

Finally, all of the proteins were purified by immobilized metal affinity chromatography (IMAC) using HisTrap HP 1–5 mL columns (GE Healthcare) in an ÄKTA pure protein purification System (GE Healthcare) upon elution by a linear gradient of elution buffer (20 mM Tris-HCl, 500 mM NaCl, 500 mM imidazole, pH 8). The production and purification of β-Gal-H6 have been described elsewhere.^25^ Recovered proteins were dialyzed against their most suitable buffer: sodium carbonate (166 mM NaHCO_3_, pH 8) for STM-H6, GFP-H6, and HSA-H6, sodium carbonate with salt (166 mM NaHCO_3_, 333 mM NaCl, pH 8) for A1-GFP-H6, B4-GFP-H6, F7-GFP-H6, BAK-GFP-H6, and H6-GFP-T22, Tris buffer (1 M NaCl, 50 mM Tris-HCl, pH 7.25) for TRX-H6-hLIF, and PBS (7.5 mM Na_2_HPO_4_, 2.5 mM NaH_2_PO_4_, 15 mM NaCl, pH 7.4) for β-Gal-H6. GFP, β-Gal, and HSA were commercially obtained as previously mentioned and subsequently dialyzed against compatible buffers: namely, sodium carbonate (166 mM NaHCO_3_, 2.8 mM EDTA, pH 8), PBS (7.5 mM Na_2_HPO_4_, 2.5 mM NaH_2_PO_4_, 15 mM NaCl, pH 7.4), and sodium carbonate (166 mM NaHCO_3_, pH 8), respectively.

### Description of Protein Modules

T22 is a cationic peptide that acts as a potent ligand of the cell surface cytokine receptor CXCR4.^26^ BH3 is a BAK protein domain (named here as BAK) that inhibits antiapoptotic cell proteins.^27^ The human leukemia inhibitory factor (hLIF) is a well-known interleukin 6 class protein that modulates cell differentiation.^28^ Thioredoxin (TRX) is an antioxidant agent that enhances protein solubility when it is used as a tag.^29^ Stefin A triple mutant (named here as STM) is a human protein usable as an inert protein scaffold.^30^ All of these protein domains were designed to be expressed from codon-optimized genes provided by GeneArt. A1, B4, and F7 ligands, architecturally arranged as nanobodies (VHHs), were obtained from a homemade phage display library based on naïve llama-derived nanobodies. The biopanning process was performed by confronting the library against the CXCR4 receptor overexpressed on SW1417 cells, as described elsewhere.^31,32^ The most specific candidates were selected, and the recombinant proteins were produced as GFP fusions (VHH-GFP-H6).

### Protein Concentration, Purity, and Integrity

The protein purity was assessed upon purification by SDS-PAGE using the TGX Stain-Free FastCast Acrylamide Kit, at 12% (BioRad). Bands were transferred to PVDF membranes by a Trans-Blot Turbo Transfer System (BioRad) and finally immunodetected by Western blot (WB) using an anti-His antibody (Ab; Santa Cruz Biotechnology) at a 1/5000 dilution. The protein integrity was determined by matrix-assisted laser desorption ionization time-of-flight (MALDI-TOF) mass spectrometry and the protein concentration by a Bradford assay.

### Protein Assembly and Disassembly

Soluble proteins at 2 mg mL^–1^ (with the exception of GFP and β-Gal, adjusted at 0.5 mg mL^–1^) in the respective optimal buffers were distributed in 50 μL aliquots. Assembly was achieved by adding a 0.22 μm filtered solution of ZnCl_2_ (stock at 400 mM and working concentrations indicated in the figures), and the mixture was gently mixed and incubated for 5 min at room temperature (RT). Disassembly was promoted by adding a solution of ethylenediaminetetraacetic acid (EDTA) at concentrations indicated in the figures, and the mixture was gently mixed and incubated for 5 min at RT. The hydrodynamic diameter of the materials was determined by dynamic light scattering (DLS) at 25 °C and 633 nm wavelength, with a Zetasizer Nano ZS instrument (Malvern Instruments Limited) using ZEN2112 3 mm quartz batch cuvettes.

### Protein Precipitation

The soluble protein was adjusted to 2 mg mL^–1^ and distributed in 250 μL aliquots (to obtain 0.5 mg of product from each aliquot, provided the precipitation would be 100% efficient). The aggregation process was promoted by adding a 0.22 μm filtered solution of ZnCl_2_ (stock at 400 mM) at concentrations depicted in the figures, and the mixture was gently mixed and incubated for 15 min at RT. Then, samples were centrifuged at 10000*g* for 10 min at RT, and the soluble fraction was collected for further quantification by a Bradford assay. The resultant isolated and precipitated microparticles were stored at −80 °C until further use and visualized, and the protein was indirectly quantified using soluble fraction measurements. Microparticles were also analyzed by DLS at 25 °C, with short measurement time periods and using 633 nm wavelength, with a Zetasizer NanoZS instrument (Malvern Instruments Limited) using ZEN2112 3 mm quartz batch cuvettes.

### Stability of Released Soluble Protein

The release of soluble protein from microparticles was promoted by the addition of 250 μL of storage buffer and subsequent resuspension at RT. Then, samples were centrifuged at 15000*g* for 5 min at RT for isolation of the soluble version. The protein size and stability were assessed at different temperatures and under thermal conditions (37 °C overnight, overnight at RT, and 4× thawing and freezing) by DLS at 25 °C and 633 nm wavelength, with a Zetasizer NanoZS instrument (Malvern Instruments Limited) using ZEN2112 3 mm quartz batch cuvettes. Protein integrity was also assessed by TGX Stain-Free electrophoretic gel (BioRad).

### Determination of Z Potential

The Z potentials (Zp) of protein materials were determined by electrophoretic light scattering (ELS) at 633 nm (25 °C), with a Zetasizer Nano ZS instrument using DTS10170 capillary cells and in the respective solubilization buffers.

### Protein Stability

Fluorescence spectra of oligomeric and building block protein forms were recorded with a Cary Eclipse spectrofluorometer (Agilent Technologies) by using a quartz cell with 10 mm path length and a thermostatic holder. The excitation and emission slits were set at 5 nm, the excitation wavelength was set at 295 nm, and the emission spectra were acquired within ranges of 310–450 nm (for HSA-H6) and 480–550 nm (for GFP-H6). The protein concentration was adjusted to 0.2 mg mL^–1^. We evaluated the thermal behavior of proteins within the range 25–50 °C in p/a of ZnCl_2_ (0.4 mM). The fluorescence intensity at λ_max_ was plotted against temperature. Additional stability determinations of oligomers and building blocks were carried out by DLS as described above, within ranges of 4–50 and 4–90 °C depending on the experimental needs.

Also, the integrity of the NPs in media without Zn was evaluated in triplicate through their size upon dialysis. GFP-H6 and HSA-H6 NPs (0.15 mL), assembled as described above, were placed in dialysis cassettes (Slide A lyzer 3.5K MWCO Dialysis cassette, Thermo Fisher Scientific) and submerged in 1 L of sodium carbonate buffer (166 × 10^–3^ M NaHCO_3_, pH 8.0) without ZnCl_2_, with agitation, at 4 °C for 30 min. Then two buffer exchanges were applied, each at RT, over 30 min and against 1 L of the same buffer. Finally, the samples were removed from the cassettes and analyzed at 25 °C with a Zetasizer Advanced Pro Blue instrument (Malvern Instruments Limited) at 633 nm, using ZEN2112 3 mm quartz batch cuvettes.

### Determination of Protein Activity

A 20 μL portion of a β-galactosidase–ZnCl_2_ mixture (diluted 100-fold with respect to DLS measurements) was mixed with 5 mM *o*-nitrophenyl galactopyranoside in a 500 μL final volume of PBS. The sample was incubated for 15 min at 37 °C, the reaction was stopped by adding 200 μL of Na_2_CO_3_ (2.8 mM), and the product amount was determined by absorbance at 420 nm (ε_420_ = 4530 M^–1^ cm^–1^) in a UV–vis spectrophotometer (Ultrospec 1000E, Pharmacia Biotech). The activity was expressed as the percentage with respect to the control without ZnCl_2_.

### Electron Microscopy

Ultrastructural morphometry of proteins at the nearly native state was assessed with two high-resolution techniques. Sample drops (5 μL) were deposited on silicon wafers (Ted Pella Inc.) for 2 min, air-dried, and immediately observed without coating with a Merlin field emission scanning electron microscope (FESEM) (Zeiss), operating at 1 kV and equipped with a high-resolution in-lens secondary electron detector. Representative images of general fields and nanostructure details were captured at two high magnifications (150000× and 400000×). Drops (5 μL) of the same samples were deposited for 2 min on 200 mesh copper grids coated with carbon, contrasted with 2% uranyl acetate for 2 min, air-dried, and observed with an H-7000 transmission electron microscope (TEM) (Hitachi) equipped with a CCD Gatan ES500W Erlangshen camera (Gatan). Representative images of general fields and nanostructure details were captured at two high magnifications (70000× and 200000×).

### Zebrafish Husbandry and Breeding

Wild type zebrafish (*D. rerio*) were kept in a recirculating aquarium with the water temperature maintained at 28 ± 1 °C. The lighting conditions were 12 h:12 h (light:dark), and adult fish were fed twice a day at a rate of 2% body weight. Ammonia, nitrite, pH, and nitrate levels were measured once a week. Ammonia and nitrite levels were kept below the detection level, and the pH was maintained between 6.8 and 7.5. The nitrate levels were maintained to be <100 mg L^–1^. For in-tank breeding, previously isolated individuals, one female and three males, were transferred to a breeding tank in the late afternoon. Embryos were collected the next morning and cultured in embryo medium (E3 medium) in a Petri dish (Deltalab). Fertilized eggs were separated from unfertilized eggs using a plastic pipet (Deltalab). All experiments involving zebrafish *(D. rerio)* were performed following International Guiding Principles for Research Involving Animals (EU 2010/63).

### Protein Uptake by Zebrafish Larvae Assessed by Fluorescent Microscopy

Groups of 15 larvae (*n* = 5/condition, 72 hpf) were distributed on 96-well plates (Thermo Fisher Scientific) with one larva per well. Wells contained 200 μL of E3 medium or 50 μgmL^-1^ of unassembled or assembled GFP-H6. *In vivo* uptake after 48 h treatment was observed in anesthetized larvae (50 ppm, MS-222) using a fluorescence stereomicroscope (Nikon SMZ800) coupled with a camera (Nikon DS-Fi2). No signs of toxicity were observed during exposure.

### Statistical Analyses and 3D-Model Visualization

The size increase factor (SIF) was calculated as the percentage of gained size upon oligomerization and statistically analyzed versus the presence or absence of the His tag or a peptidic ligand, the type of scaffold (GFP-based or others), or the number of histidine residues in the primary structure (more or less than 14). For all collations, an initial variety of normality and log normality tests (Anderson–Darling, D’Agostino & Pearson, Shapiro–Wilk and Kolmogorov–Smirnov) were used to stablish data normal distribution. Both *t* tests (single comparisons) and one-/two-way ANOVA (multiple comparisons) were used to determine the significance for parametric data, and the Mann–Whitney test was used for nonparametric data. Statistics in all data sets were performed at least in triplicate (*n* = 3), expressed as mean ± standard error of the mean (*x̅* ± SEM) and significance (*p ≤ 0.05 or *p < 0.001, respectively) in comparison to the control group. HSA and GFP models were generated using the Robetta web server^33^ and visualized in UCSF Chimera.^34^

## Results and Discussion

Exploiting the universal H6 tag as an architectonic agent (Figure 1A) would allow the implementation of a fully transversal platform for the *on-demand* fabrication of protein nanoparticles. This would be feasible, provided divalent aggregation mediated by cations could generate regular oligomers as stable and functional intermediates in the clustering process that finally renders amorphous microparticles (Figure 1B). This possibility was initially assessed by testing two structurally dissimilar proteins commonly used in research laboratories: namely, HSA and GFP (Figure 1C). The H6-tagged forms of these proteins (HSA-H6 and GFP-H6, respectively) showed hydrodynamic sizes between 5 and 6 nm, compatible with their unassembled forms (Figure 1D). However, when these proteins were incubated with equimolar amounts of Zn^2+^ (0.4 mM), relatively monodisperse oligomers were observed of around 15 and 10 nm, respectively (Figure 1D), with a similar and slightly negative Z potential (Figure 1D, bottom). The resulting assemblies that were not built up by the wild-type protein versions lacking H6 (Figure 1E) were disassembled by the chelating agent EDTA (Figure 1E), demonstrating the relevance of the metal in the assembly process. The occurrence of several His residues in the native sequence of the proteins (Figure 1C) was not sufficient to support cation-dependent assembly, suggesting that a local His clustering was required to promote stable protein–protein contacts. Zn^2+^ concentrations above 0.4 mM resulted in a clear tendency to form large aggregates instead of regular oligomers, approaching the micrometer scale (Figure 1F). This observation supported the hypothesis that protein oligomers organized into regular NPs are intermediates in a process finally leading to the formation of amorphous MPs (Figure 1B). While protein assembly as nanoparticles would indeed be triggered by equimolar amounts of the ion (calculated with regard to the six histidine residues in the H6 tails), multivalent contacts with excess ions would induce protein aggregation as microscale complexes (Figure 1A,B). Such aggregation at higher concentrations of Zn^2+^ indicates that the amounts of ions used for NP formation were not saturating all of the available histidine residues in the proteins.

Interestingly, the Zn-based NPs were stable, once formed, in Zn^2+^-free media, as demonstrated by DLS for both HSA-H6 and GFP-H6 (Figure 1G). This observation indicated that the cation used for assembly formed robust interactions with His-tagged proteins and that such interactions were not resulting from an equilibrium with free cations in the media. This fact was particularly interesting, envisaging the potential *in vivo* applications of this category of material or, in general, uses in environments whose ionic composition cannot be controlled.

**Figure 2:**
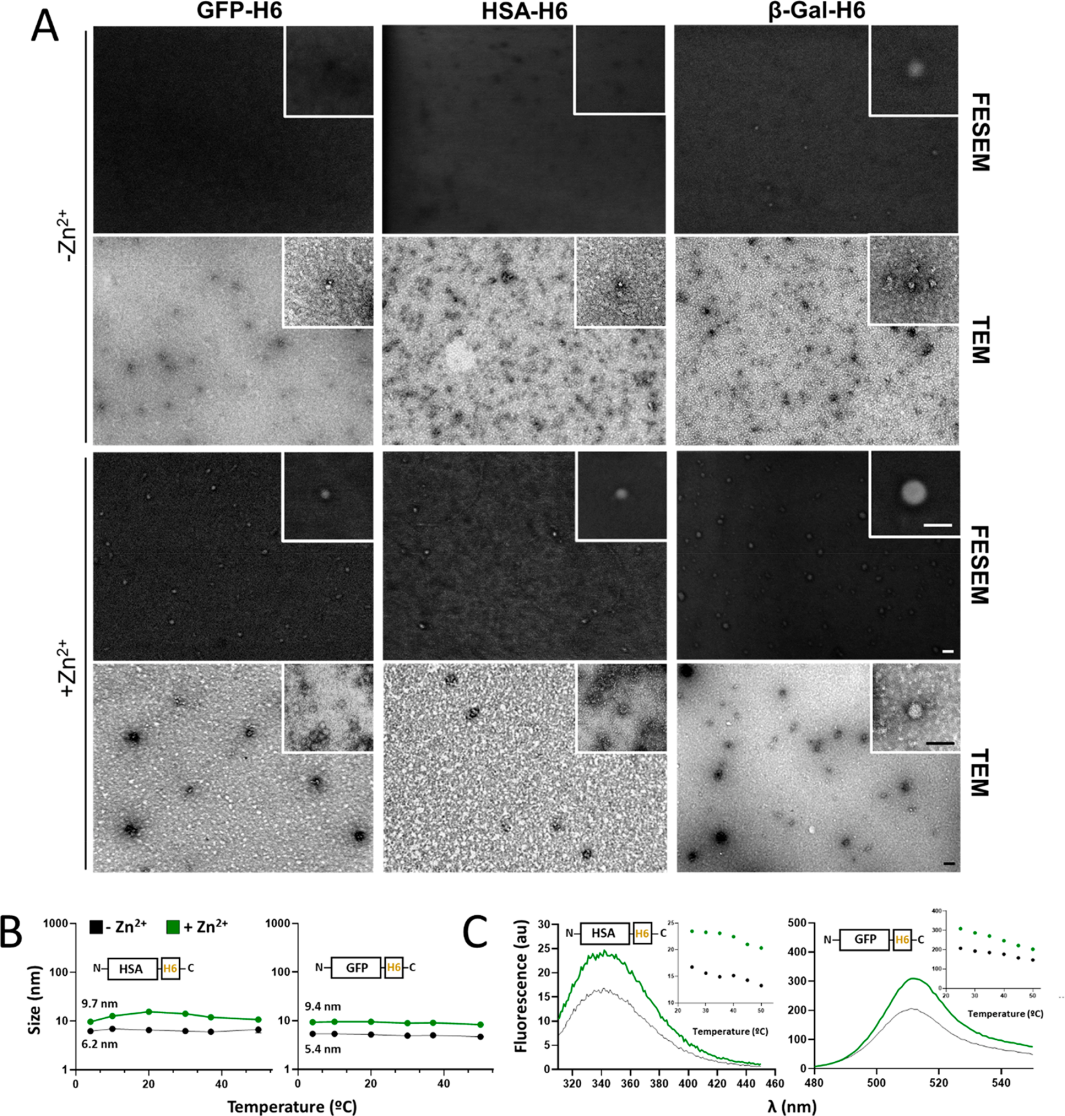
Structural analysis of Zn^2+^-mediated oligomers. (A) Representative high-resolution electron microscope images (TEM and FESEM) of single monomers without Zn (−Zn^2+^) and well-formed nanoparticles (+Zn^2+^). Scale bar size: 25 nm. (B) Stability of the materials upon an increase in temperature (from 4 to 50 °C) in the presence (assembled, green) or absence (unassembled, black) of Zn^2+^ (at 0.4 mM). (C) Fluorescence emission spectra in the presence (assembled, green) or absence (unassembled, black) of Zn^2+^ at 25 °C and a thermal profile of the fluorescence peak (au) from 25 to 50 °C (inset).

TEM and FESEM analyses allowed visualizing the formed oligomers as discrete NP entities with pseudospherical geometries, with sizes compatible with those observed by DLS (Figure 2A). When they were tested for stability, the assembled materials maintained the thermal resistance of the building blocks (Figure 2B). The fine analysis of protein fluorescence revealed differences in the emission profiles of building blocks and NPs (Figure 2C), which indicated structural rearrangements during oligomerization as previously observed in other assembly setups.^35^ Such a structural profile was indicative of conformational adaptation to the partner subunits in the oligomer, and it was maintained over a wide temperature range (Figure 2C, insets). This fact indicated again that, once assembled, the protein NPs were structurally stable.

**Figure 3:**
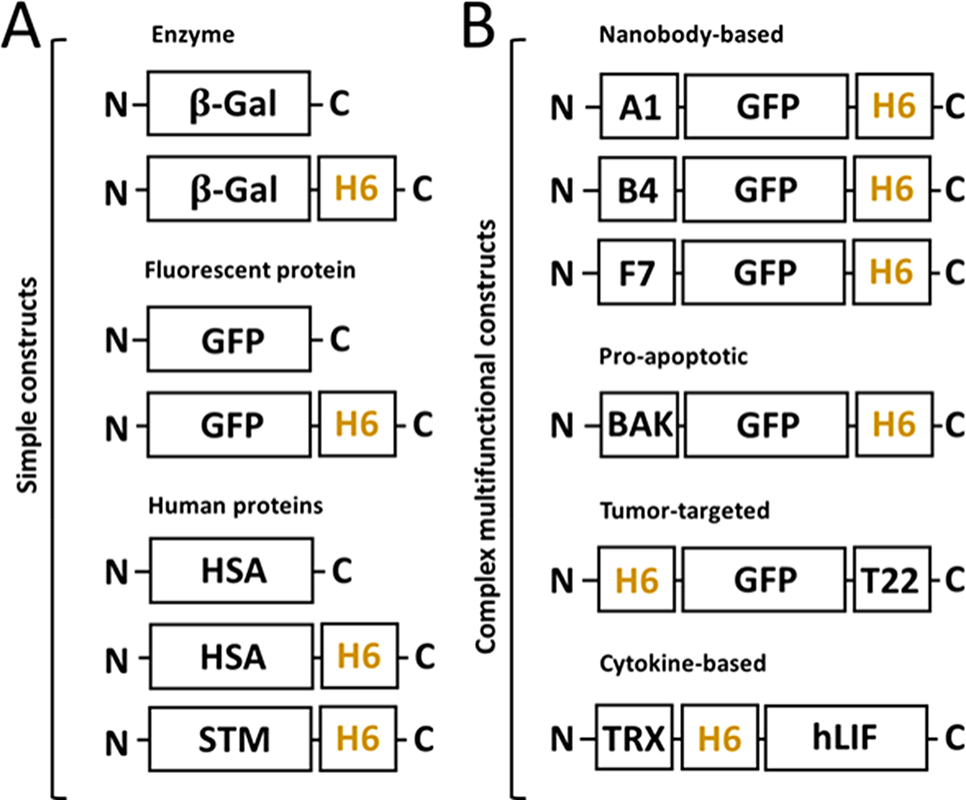
Comparative modular organization of the proteins tested in the study. (A) Representation of moderately engineered constructs with or without an H6 tag, categorized depending on their nature (enzyme, fluorescent, or human-based). (B) Representation of complex modular constructs developed as targeted protein drugs, categorized depending on the nature of the main functional domain (nanobody, proapoptotic, tumor-targeted, or cytokine-based). Relative box sizes are only indicative.

**Figure 4:**
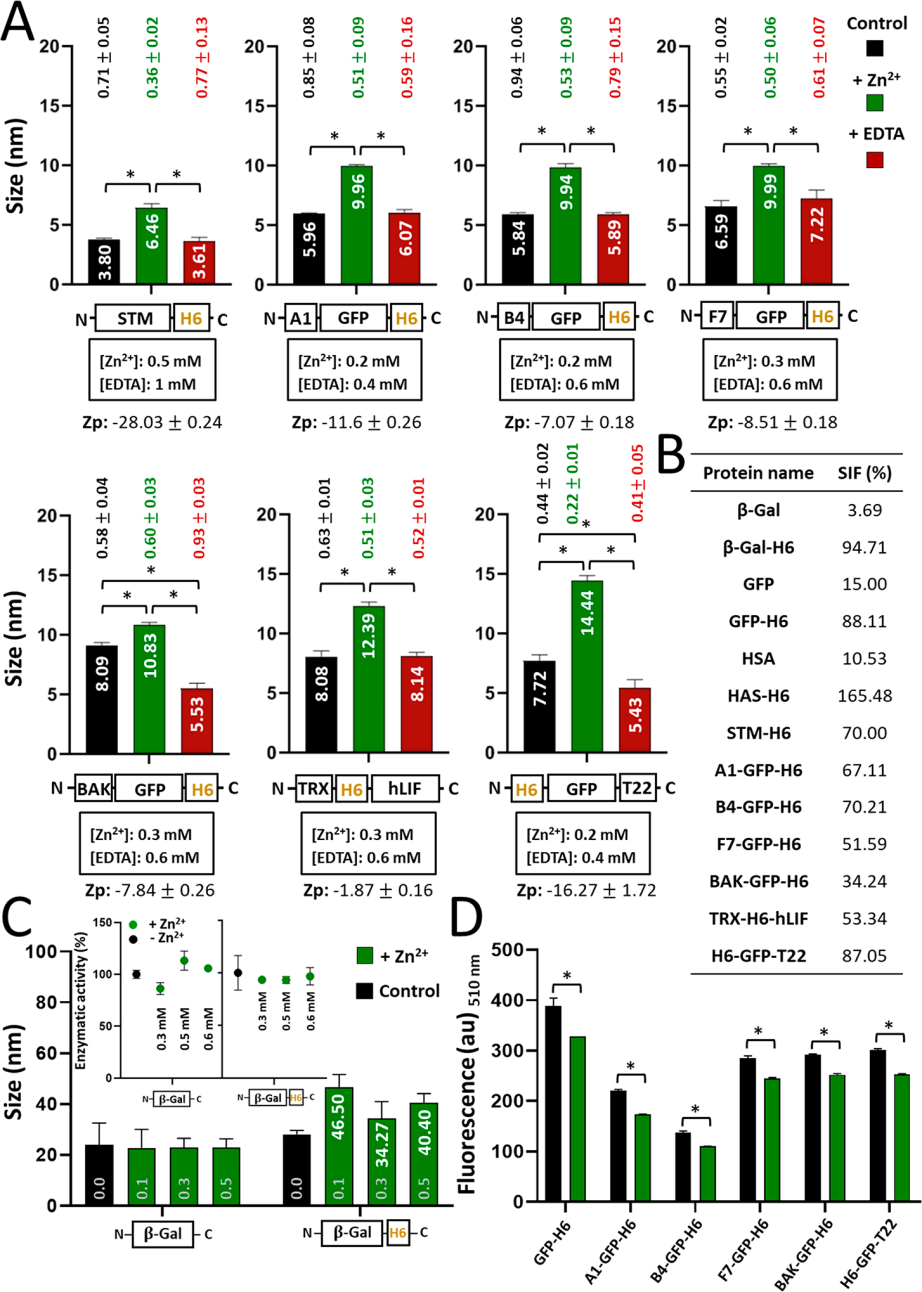
Oligomerization and biological activity of protein materials. (A) Hydrodynamic size of assembled (in the presence of Zn^2+^, green bars) and further disassembled (with the addition of EDTA, red bars) proteins. The concentrations of Zn^2+^ and EDTA are depicted below the protein names. Protein samples without chemical agents are expressed as black bars (control). Pdi values are indicated above all samples with the respective colors, and the Zp values of samples containing Zn^2+^ are shown at the bottom expressed in mV. (B) Size increase factor (SIF), referring to the percentage of size increase observed in cation-treated proteins with reference to the unassembled material. (C) Assembly profiles of β-Gal with or without an H6 tag, at increasing Zn^2+^ concentrations (namely 0.1, 0.3, and 0.5 mM, depicted in gray). White numbers indicate the hydrodynamic sizes in nm. In the inset, the absence of the effect of Zn^2+^ on the enzymatic activity of β-Gal and β-Gal-H6. (D) Fluorescence emission measured at 510 nm of GFP-based proteins expressed in arbitrary units (au). The legend is the same as that in (C). Data sets are expressed as *x̅* ± SEM with at least *n* = 3, and statistical significance is achieved when *p < 0.05 for (A)–(C) and *p < 0.001 for (D).

**Figure 5:**
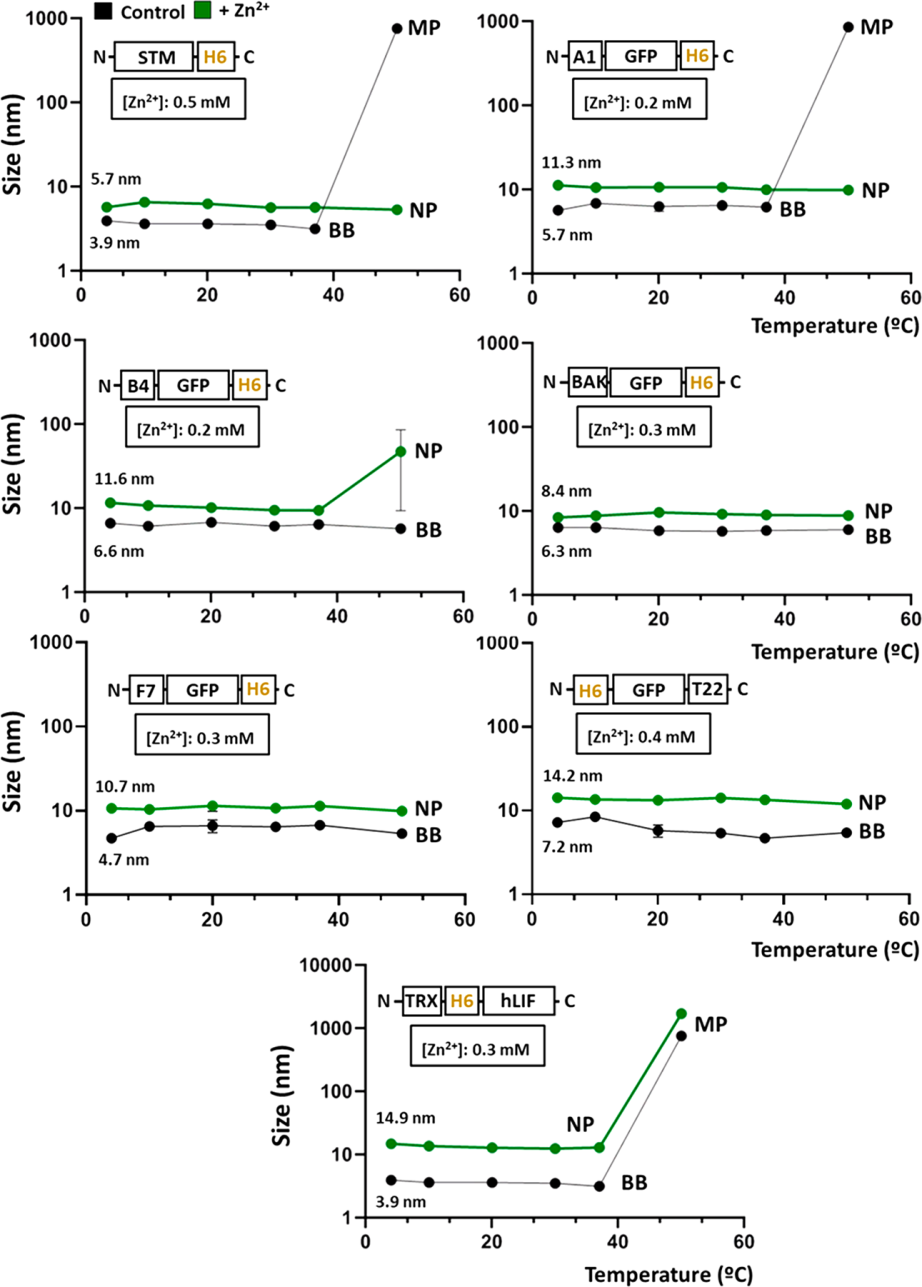
Oligomer stability upon thermal exposure. Size analysis of protein constructs on increases in temperature (from 4 to 50 °C) in the presence (green) or absence (black) of specific amounts of Zn^2+^ as indicated in boxes. Data sets are graphically named according to the material size as building blocks (BB), nanoparticles (NP), or microparticles (MP) and expressed as *x̅* ± SEM with at least *n* = 3.

At this point, and to confirm the transversal nature of the Zn^2+^-mediated oligomerization and the robustness of this approach, additional H6-tagged proteins used in the laboratory, including natural and largely engineered modular species of different origins, were tested (Figure 3). These proteins exhibit different functionalities, and consequently, they are suitable for different applications *in vitro* and *in vivo*. For this last use, it is important to obtain nanostructured drugs with dimensions above the renal clearance threshold (around 6–7 nm),^36^ which cannot be reached by the wild-type monomeric or dimeric proteins. Protein NPs offer further advantages over their plain protein versions, including enhanced proteolytic stability,^37^ improved targeting,^38^ and full exploitation of the enhanced permeability and retention (EPR) effect,^39^ apart from the whole set of appealing physical properties associated with nanoscale materials (namely, their particular behavior between liquids and solids and their elasticity and other mechanical features^40^). As observed, all tested His-tagged proteins, but not their counterparts devoid of H6 (when available for testing), increased in hydrodynamic size in the presence of equimolar (0.2–0.5 mM) Zn^2+^ concentrations (Figure 4A,B). The assembled materials usually reached around 10 nm in size, had relatively low polydispersion indexes, and were efficiently disassembled by EDTA (Figure 4A). Interestingly, assembly occurred irrespective of the localization of H6, at the N terminus (H6-GFP-T22), at the C terminus (most of the modular proteins) or in an internal accommodation site as a linker between functional domains (TRX-H6-hLIF). Also, a structurally complex and large tetrameric enzyme, the *E. coli* β-galactosidase,^41^ showed the same oligomerization profile upon exposure to the metal. The tagged version of the enzyme formed self-organized supramolecular complexes of around 40 nm in size (Figure 4C). This occurred in the absence of significant modifications in the enzymatic activity mediated by the ion, irrespective of the presence or absence of the H6 tag (Figure 4C, inset). Despite the fact that the wild-type β-galactosidase contains multiple His residues in its primary sequence,^41^ these were not effective for promoting oligomerization at the tested Zn^2+^ concentrations. On the other hand, ion-mediated formation of NPs did not abort green fluorescence emission in any of the GFP-containing proteins tested here (Figure 4D), indicating the maintenance of the essential conformational patterns of the building block proteins and therefore their global functionality. Again, all of these materials were proved to be stable (Figure 5). In fact, in the case of STM and A1-based constructs, oligomerization conferred clear protection in front of thermal aggregation in comparison to the respective unassembled building blocks (Figure 5).

**Figure 6:**
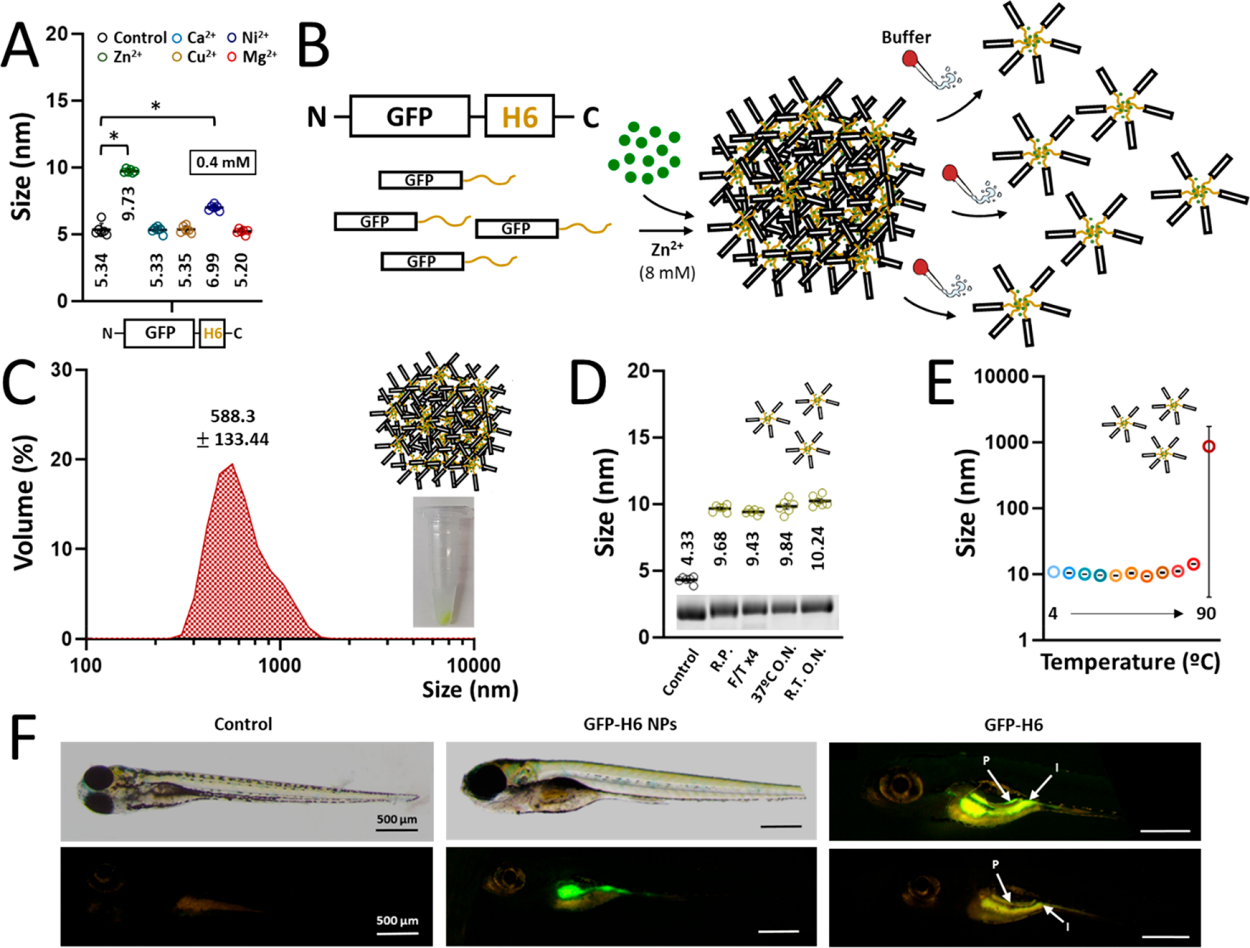
Manufacturing artificial GFP-H6 MPs and stability analysis of released NPs. (A) Assembly of GFP-H6 upon the addition of different divalent cations (Zn^2+^, Ca^2+^, Cu^2+^, Ni^2+^ and Mg^2+^) at 0.4 mM. Numbers refer to mean size. (B) Schematic representation of MP formation upon addition of excess (8 mM) Zn^2+^ and subsequent protein NP release after resuspension with protein storage buffer. (C) Hydrodynamic size (nm) of GFP-H6 microscaled particles at 8 mM Zn^2+^. The inset picture shows the obtained fluorescent pellet after particle formation. (D) Hydrodynamic size (nm) of released soluble GFP-H6 NPs after MP resuspension under different thermal conditions. R.P. refers to released protein. (F/T x4) refers to freezing and thawing the sample for four cycles. 37 °C O.N. refers to protein incubation at 37 °C overnight. R.T. O.N. refers to protein incubation at RT overnight. The inset shows a TGX gel under each experimental condition. **E.** Hydrodynamic size (nm) of released soluble NPs at increasing temperatures (4, 10, 20, 30, 37, 50, 60, 70, 80, and 90 °C). (F) *In vivo* uptake experiment in 5 dpf zebra fish larvae. Larvae were immersed in 50 μgmL^-1^ of GFP-H6, either as building blocks or as NPs and thus exposed for 48 h. In the panels with quantitative data, data sets are expressed as *x̅* ± SEM with at least *n* = 3, statistical significance (*) is achieved when *p* < 0.05, and the peak value is depicted when it applies.

To explore the versatility and the *in vivo* applicability of the type of NPs generated in the present study, we first tested if divalent cations different from Zn^2+^ could also drive NP formation. As was observed (Figure 6A), Ni^2+^, but not Ca^2+^, Cu^2+^, and Mg^2+^, at the tested concentration (0.4 mM) was also able to promote oligomerization of GFP-H6. Nevertheless, Zn^2+^ was clearly the most efficient linker in the process, rendering larger materials. Interestingly, if the H6-dependent formation of NPs is an intermediate step in the formation of MPs (Figure 6B), it would be interesting to know if MPs could act as a reservoir of NPs for potential clinical applications. This potential applicability of protein MPs as protein drug depots for clinical uses has been demonstrated for a category of natural amyloids called bacterial inclusion bodies^42^ and for artificial versions of such natural materials,^43^ generated by clustering, either *in vivo* or *in vitro*, respectively, of receptor-targeted protein drugs. The application of 8 mM Zn^2+^ to soluble GFP-H6 resulted in polydisperse MPs peaking at around 0.5 μm but reaching sizes of over 1 μm (Figure 6C). These materials, insoluble and fully fluorescent (Figure 6C, inset), spontaneously released NPs ranging between 9 and 10 nm under different incubation conditions (Figure 6D), indistinguishable in size from those generated by the straightforward addition of 0.4 mM Zn^2+^ to soluble GFP-H6 (Figure 1D–F). The release of such NPs, which show great thermal stability (Figure 6E), confirmed that these materials are intermediates in the process of MP formation (Figure 6B), which is fully reversible under physiological conditions.

Finally, a full test of the clinical uses of the type of protein NPs generated here is beyond the scope of the present study. However, we wanted to check the stability of the NPs *in vivo* and the maintenance of their nanoscale structure in biological interfaces, using GFP-H6 NPs as a convenient model. For that, zebrafish larvae (5 dpf) were imaged after 48 h of *in vivo* exposure to GFP-H6, either in a soluble form or as assembled NPs (both at 50 μg/mL) to evaluate their uptake. We were particularly interested in checking the stability of the NP version and also in evaluating whether the nanoscale size could confer nanomaterial properties to the soluble protein and thus limit the broad diffusion in biological tissues expected for a plain unassembled polypeptide. Both biomaterials were taken up orally by the larvae (Figure 6F). While NPs accumulated uniquely in the intestine, indicative of an enhanced tissue retention expected for a nanostructured material,^44^ the soluble version dispersed through the intestine and pancreas with background in other tissues (Figure 6F). Also, the NP version rendered a more intense fluorescence signal (Figure 6F), altogether indicative of the high stability of NPs and the maintenance of their distinctive structure and fluorescent emission once in the body.

**Figure 7:**
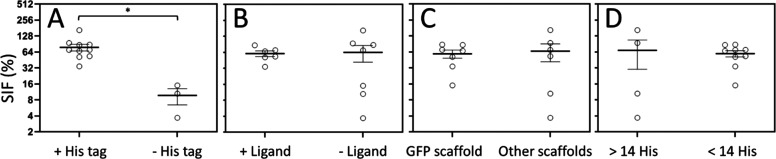
Effect of intrinsic protein parameters on protein self-assembly. Statistical analysis comparing the SIF upon nanoparticle formation versus intrinsic protein properties, namely the presence or absence of H6 (A), the presence or absence of targeting peptides (B), the type of protein scaffold (C), and the number of histidine residues in the native protein (D). Data sets are expressed as scattered dot plots; *x̅* ± SEM with at least *n* = 3, and statistical significance is achieved when *p < 0.05. SIF is the size increasing factor.

This whole set of data proved that the addition of a simple H6 tag to simple or complex protein constructs (Figure 3) allowed their controlled oligomerization into regular and reproducible nanoscale complexes, following an assembly concept of generic applicability based on divalent cation coordination. None of the main biochemical traits of the model proteins, such as the presence of a naturally interacting peptide ligand (such as T22 used for cell targeting), the type of scaffold, or the number of His residues in the native protein (excluding H6), had any influence on the assembly process (Figure 7). His residues clearly need to be clustered such as in a His-rich tag for protein assembly as nanoparticles, and consequently, oligomerization of proteins in the presence of Zn^2+^ due to naturally occurring His residues in their sequence is not observed here and is assumed to be improbable. The single functional domains present in the building blocks preserve their biological activity once they are assembled into NPs, as tested by the GFP specific fluorescence of all GFP-containing NPs in comparison to the unassembled building blocks (Figure 4D). The experimental data showed that oligomers kept the emission capabilities of the original protein, although a tendency toward a moderated reduction was regularly observed. This could be due to quenching of the GFP fluorophore by protein–protein contacts, as previously suggested for similar GFP-based oligomers.^45,46^ Interestingly, the stability of all multimeric constructs was very high. The experiments of thermal challenge (Figures 2, 5, and 6E) showed that most of the tested constructs were thermally more stable than the equivalent building block versions, proving the robustness of the resulting materials, as expected from the protein assemblies.^46^ On the other hand, the clustered Zn^2+^ cation, although removable by EDTA (Figure 1E), appears to not be released from the oligomers by mere dilution, as the NPs remain stable upon dialysis against a Zn-free buffer (Figure 1G), again pointing out the structural robustness of the formed material. Interestingly, the protein NPs generated here are proved to be stable intermediates in the formation of MPs (Figure 1A). Despite the mechanical stability of the MPs (Figure 6C), the clustering process appears to be fully reversible under physiological conditions (Figure 6B,D). In this context, the MPs formed at high ion concentration released NPs that were indistinguishable from those directly formed at low ion concentrations (Figure 6D) and that are highly stable when they are challenged with high temperatures (Figure 6E). The high stability of NPs generated with clustering H6-tagged proteins has also been demonstrated *in vivo* using a fluorescent GFP-H6 material, which in the oligomeric but not in the soluble form is retained in the gut upon oral administration (Figure 6F).

Other protein oligomerization platforms for full-length proteins have been described that allow the assembly of desired polypeptides, either by the incorporation of natural oligomerization domains^13,47^ or by the combination of several types of cationic peptides that are placed at particular accommodation sites, to promote cross-molecular interactions.^48^ The technology proposed here is based on the simple natural observation that divalent cations, Zn^2+^ among others, drive the assembly of proteins into amyloidal structures,^49−51^ protein reservoirs, and scaffolds,^18,23,52−55^ in different biological contexts. The addition of a H6 tag to recombinant proteins is a common procedure to simplify their purification,^56−58^ and therefore, most of the proteins are already suitable to undergo the assembly process described here. Such tag incorporation is the only required engineering process that, being simple and versatile, involves a minor modification of the protein properties, in most cases without a noticeable effect on their biological activity. The potential use of metals (or mixtures) others than Zn (such as Fe) in their cationic forms could, in addition, confer supplementary properties to the H6-based protein NPs such as their magnetic manipulation, which could be of particular interest in specific clinical contexts.^59^

The transversal applicability of this protein assembly approach, as demonstrated here by a broad set of unrelated proteins (Figures 1 and 3) and by the sole dependence of the H6 tag among the tested parameters (Figure 7), opens the door to the general generation of nanostructured versions of proteins of interest. In this regard, protein oligomers, built in with intrinsically biocompatible and biodegradable macromolecules, are very interesting in comparison to categories of materials such as metals, ceramics, lipids, and carbon nanotubes, which pose concerns regarding toxicity and permanence in the environment.^60−66^ On the other hand, in contrast to other nanomaterials, protein NPs do not recruit protein corona upon systemic application, avoiding a critical bottleneck in the use of nonprotein nanomaterials as drug delivery systems.^67,68^ This is also important because proteins are a unique category of drugs that allow their self-assembly and self-delivery in the absence of chemically heterologous drug carriers.^69^ Then they fulfill, in that way, a major concept in nanomedicine aimed at preventing toxicities and increasing efficacy: that is, removing nanoscale vehicles from drug delivery systems.^70^ The chemical homogeneity of proteins as increasingly approved biopharmaceuticals^71^ in combination with the use of physiological linkers such as natural cations^17^ makes them highly appealing, especially if they can be manipulated to gain stable nanoscale-sized functionalities, as demonstrated here (Figures 1, 2, and 6). More than 400 protein products have been approved as human drugs^71^ as the result of scalable and green biofabrication processes,^72^ again in contrast with less environmentally friendly nanodrug fabrication procedures.^73^ Some of these protein pharmaceuticals might benefit from their administration as nanostructured multivalent forms, since we show here that the nonspecific diffusion capabilities in living tissues are restricted in such a version (Figure 6F). For cell-targeted protein drugs, the multivalent display of cell-receptor ligands has been also proved to enhance binding, internalization and delivery in target cells.^74^ Also, it must be noted that chemical linkers used to generate antibody–drug conjugates and other drug constructs represent an added toxicological risk to the final product.^75−78^

Finally, the use of H6 as a clustering agent might be controversial in a clinical context due to the potential immunogenicity of H6. As a matter of discussion, no evidence of H6-linked immunopathology is available. In contrast, a simple BLAST search in the human proteome for H6 (not shown) reveals a significant list of outputs, including intracellular and extracellular proteins such as Q6UXD1,Q70CQ2, O00555, Q4VCS5, O43497, and Q9Y566 (Uniprot codes). Of course, since there is evidence which points out that other human His-rich domains might be also useful to drive protein oligomerization, fully humanized alternatives might be further explored with regard to fully biocompatible protein materials,^38,56^ if this is proved to be convenient for particular cases.

## Conclusion

On the bases of the presented data, we propose the use of divalent cations as a universal trigger of His-tag-based protein oligomerization into the nanoscale, through a simple procedure that can be applied on demand to any His-tagged polypeptide. Other strategies based on structural selection, ensuring periodicity between particular amino acids (Glu, Cys, Asp, and His^79^) or through the incorporation of artificial amino acids with high affinity for cations,^80^ are also addressed to facilitate the formation of oligomeric structures. However, none of them refer to the use of a His tag as an architectonic agent for NP formation. This fact and the universal use of polyhistidines in protein production give a distinctive and totally innovative character to this new platform, which is based on fully environmentally friendly, sustainable processes supported by physiological amounts of cations present in living systems. Notably, the number of protein drugs in the market is steadily increasing^71^ and this tendency will probably intensify in the future. The administration of nanoscale functional versions of otherwise monomeric drugs would provide the beneficial multivalent presentation of molecular ligands, enhanced endocytosis, and improved stability, biodistribution, and functionality. In addition, our approach fulfills the rising nanomedical concept of carrierless (or vehicle-free) nanomedicines, in which the drug itself self-organizes as nanoparticles in the absence of any heterologous vehicle.^70^ The simplicity of Zn^2+^-supported assembly^17^ that uses cation doses much below the toxic threshold^17,81^ allows for the immediate application of this technology in industry and biomedicine and the conversion into functional nanostructures of the huge catalogue of already existing His-tagged proteins.

## Abbreviations

β-Gal: β-galactosidase
BB: building block
DLS: dynamic light scattering
EDTA: ethylenediaminetetraacetic acid
FESEM: field emission scanning electron microscopy
GFP: green fluorescent protein
H6: hexahistidine
hLIF: human leukemia inhibitory factor
HSA: human serum albumin
IMAC: immobilized metal affinity chromatography
IPTG: isopropyl β-d-1-thiogalactopyranoside
NP: nanoparticle
MP: microparticle
Pdi: polydispersion index
SEM: standard error of the mean
STM: stefin A triple mutant
TEM: transmission electron microscopy
TRX: thioredoxin
VHHs: nanobodies
